# Comparative adaptability of 307 *Saccharomyces cerevisiae* strains from winemaking and Mantou fermentation

**DOI:** 10.3389/fmicb.2025.1581370

**Published:** 2025-04-11

**Authors:** Cairong Su, Hui Wang, Yiming Jia, Wenhua Wang, Xueli Ma, Peijie Han, Lixia Zhu

**Affiliations:** ^1^College of Food Science and Engineering, Tarim University, Alar, Xinjiang, China; ^2^State Key Laboratory of Mycology, Institute of Microbiology, Chinese Academy of Sciences, Beijing, China; ^3^Production & Construction Group Key Laboratory of Special Agricultural Products Further Processing in Southern Xinjiang, Alar, China

**Keywords:** *Saccharomyces cerevisiae*, population adaptability, dough, grape juice, stress tolerance

## Abstract

Domesticated *Saccharomyces cerevisiae* is one of the most significant microbial populations in human civilization due to its remarkable diversity and high adaptability to human environments. However, the adaptability mechanisms underlying this population ecological behavior remain unclear. This study explored the adaptive behaviors of *S. cerevisiae* strains from the Wine and Mantou genetic lineages under both artificial stress conditions and natural or near-natural environments. A total of 307 diploid *S. cerevisiae* strains were analyzed, including 169 strains derived from grape sources and 138 from sourdough sources. Various stress factors, including sodium chloride, tannins, ethanol, pH, temperature, and sulfur dioxide (SO_2_), as well as different substrates (natural grape juice, simulated grape juice, and simulated dough), were applied to evaluate adaptability. The results demonstrated that Wine population exhibited superior performance in grape juice environments, characterized by higher CO_2_ production. The biomass of both the Wine and Mantou populations in the simulated dough was significantly higher than that in the simulated grape juice. In the simulated grape juice environment, the adaptability of the Wine population was significantly superior to that of the Mantou population. In contrast, in the simulated dough environment, the Mantou population exhibited better adaptability than the Wine population. Furthermore, Wine population displayed higher tolerance to ethanol, extreme temperatures, tannins, and sodium chloride in YPD medium compared to Mantou population. Diploid strains also exhibited greater stress tolerance than haploid strains. These findings offer valuable insights into the distinct adaptive mechanisms of domesticated *S. cerevisiae* lineages.

## 1 Introduction

Recent research on the evolutionary phylogeny of *S. cerevisiae* populations has identified two major groups: wild and domesticated (Duan et al., 2018). The domesticated group is further classified into sub-lineages specifically adapted to liquid fermentation environments (e.g., the Wine lineage) and those adapted to solid fermentation environments (e.g., the Mantou lineage) (Duan et al., 2018; Han et al., 2021; Tellini et al., 2024). Domesticated *S. cerevisiae* is one of the most important microbial populations for human civilization. The domesticated population plays a crucial role in the production of bread and wine, exhibiting extensive diversity and strong adaptability to environments such as grape juice and wet dough. Grape juice introduces a range of stress factors that challenge *S. cerevisiae* survival, including high polyphenol content, acidity, osmotic pressure, and sugar levels, as well as low pH, added sulfur dioxide (SO_2_), controlled low temperatures, elevated temperatures during natural fermentation, and high alcohol content (Gao et al., 2022; García-Ríos and Guillamón, 2019; Gobert et al., 2019; Lin et al., 2024). Despite these challenges, *S. cerevisiae* remains the dominant microorganism in fermentation processes due to its exceptional adaptability. In starch-rich environments, such as with a nearly neutral pH, *S. cerevisiae* thrives by efficiently metabolizing key nutrients like maltose (Aydın et al., 2022; Lahue et al., 2020). This suggests that *S. cerevisiae* has developed distinct adaptive strategies, enabling it to thrive in various ecological niches, which has resulted in the formation of specialized lineages (Han et al., 2021; Tellini et al., 2024; Wang et al., 2012). Based on the excellent fermentation properties of indigenous *S. cerevisiae* in grape juice and dough fermentation, many researchers have selected potential strains from natural habitats for industrial applications (Mudoor Sooresh et al., 2023; Parapouli et al., 2020; Zhang et al., 2021). The application of individual strain fermentation and limited strain combinations has significantly advanced the industrial production of wine and bread (de Gioia et al., 2022; Ojeda-Linares et al., 2022; Rădoi-Encea et al., 2023). However, this approach has limitations to rich the quality of fermented foods, and to balance between tradition and innovation in contemporary winemaking (Gardner et al., 2023; Zampi and Ranfagni, 2024). Microbial populations, compared to individual strains, demonstrate a greater capacity to endure intense selective pressures (Swamy and Zhou, 2019). They undergo adaptive selection, characterized by mutations that confer a survival advantage in specific new environments (Heidenreich, 2007; Massoud and Zoghi, 2022; Tellini et al., 2024). These mutations reflect stable evolutionary adaptations to environmental changes. For example, in grape juice and dough, *S. cerevisiae* relies on the collective behavior of its population to carry out enological functions. Effectively managing this population during natural fermentation has increasingly become a central focus of research (Yi et al., 2024; Zhang et al., 2021).

Currently, research on the adaptability of *S. cerevisiae* populations derived from limited ancestors’ strains is primarily conducted in laboratory evolution (Caspeta and Nielsen, 2015; Godara and Kao, 2021; Randez-Gil et al., 2020). Through serial propagation, the resulting populations exhibit genetic variations, phenotypic changes, and alterations in survival capabilities as they adapt to the predefined environment (Betlej et al., 2020; Godara and Kao, 2021; Zheng and Wang, 2015). There are also many studies that focus on single strains or a limited number of strains cultivated under specific artificial conditions (Lázari et al., 2022; Mudoor Sooresh et al., 2023). This approach seeks to elucidate the mechanisms by which microorganisms adapt to specific natural environments. However, it may inadequately represent the adaptability of natural *S. cerevisiae* populations under actual conditions, potentially leading to discrepancies.

The complexity of natural environments, such as grape juice ecosystems, has led many scholars to propose that *S. cerevisiae* populations from different regions display distinct “terroir characteristics” (Alexandre, 2020; Pretorius, 2020; Rădoi-Encea et al., 2023). However, some researchers contend that this assumption is unfounded (Marsit and Dequin, 2015; Šuranská et al., 2016). In nature, *S. cerevisiae* exhibits both haploid and diploid life forms (i.e., MAT-a and MAT-α mating types, with diploid forms being predominant) (Zhang et al., 2017). Although the molecular mechanisms driving the adaptive evolution of various evolutionary lineages and ploidy types of *S. cerevisiae* are well studied, our understanding of population-level adaptive behaviors under real or near-real environmental conditions remains limited.

This study examined the population-level adaptive behaviors of *S. cerevisiae* in 169 Wine strains and 138 Mantou strains collected from regions along the Silk Road (within China) under multi-stress conditions in both native and non-native habitats. The findings reveal the adaptability of domesticated populations, shedding light on potential intrinsic patterns and providing novel strategies and perspectives for industrial applications.

## 2 Materials and methods

### 2.1 Experimental strains

This study utilized a total of 310 yeast strains, including 307 wild strains. Among these, 169 strains of *S. cerevisiae* were from the Wine population of grapes (SCP), and 138 strains were from the Mantou population of sourdough (SCJ). Specifically, 149 strains originated from Xinjiang (XJ), while 158 strains were sourced from regions outside Xinjiang (NXJ). The phylogenetic analysis revealed that these strains belonged to various lineages, including CHN-VI/VII, Daqu/Baijiu, CHN-VIII, Mantou 3, West African cocoa, Milk/Cheese Milk, European Wine, Mantou 5, Huangjiu, Mantou 7, and Mosaic lineages (e.g., Ecuador Beer 8. Mixed origin, Belgium Human/Clinical 8. Mixed origin, France Human/Clinical 8. Mixed origin, Ethiopia Honey Wine ADY/Mixed, China Plant 8. Mixed origin, China Commercial ADY, Slovakia Water 19. Mixed origin, China Mantou ADY). All wild strains were confirmed to be diploid through ploidy analysis (Supplementary Table 1) (Xiao et al., 2004; Xue et al., 2012). From each lineage, 2–3 *S. cerevisiae* strains with distinct geographical origins were selected, resulting in a total of 25 strains for the construction of wild-type haploid strains (Xiao et al., 2004; Xue et al., 2012). Additionally, haploid strains BY4741 (MAT-a) and BY4742 (MAT-α) (obtained from the Institute of Microbiology, Chinese Academy of Sciences), as well as the reference strain S288C (purchased from Biosune Biotechnology), were used as reference strains.

### 2.2 Reagents and medium

Reagents: Sterile 30% glycerol, sodium chloride, tannic acid, sodium thiosulfate, and snail enzyme (100 mg/mL).

YPD liquid medium was prepared by dissolving 10 g of yeast extract, 20 g of glucose, and 20 g of peptone in distilled water. The solution was sterilized at 121°C for 30 min.

Grape juice was freshly pressed and stored at −20°C for subsequent use.

Synthetic grape juice (SG) medium composition:

The SG medium was prepared with the following components (per liter): glucose (60 g), fructose (60 g), dipotassium hydrogen phosphate (1.14 g), magnesium sulfate heptahydrate (MgSO_4_⋅7H_2_O, 1.23 g), calcium chloride dihydrate (CaCl_2_⋅2H_2_O, 0.44 g), potassium bitartrate (cream of tartar, 2.5 g), malonic acid (3 g), citric acid (0.2 g), ammonium hydrogen phosphate [(NH_4_)_2_HPO_4_, 0.4 g], vitamin solution (10 mL), amino acid solution (10 mL), trace element solution (1 mL), and ergosterol (12.5 mg).

Vitamin solution (mg/L): the vitamin solution contained inositol (100 mg), pyridoxine hydrochloride (Vitamin B6, 2 mg), niacin (2 mg), calcium pantothenate (1 mg), thiamine hydrochloride (Vitamin B1, 0.5 mg), para-aminobenzoic acid (0.2 mg), riboflavin (Vitamin B2, 0.2 mg), biotin (0.125 mg), and folic acid (0.2 mg).

Amino acid solution (mg/L): the amino acid solution included alanine (5.9 mg), arginine (137.3 mg), asparagine (36.5 mg), aspartic acid (23.1 mg), glutamine (48.7 mg), glutamic acid (30.8 mg), glycine (4.1 mg), histidine (45.8 mg), isoleucine (24.1 mg), lysine (61.5 mg), methionine (20 mg), phenylalanine (11.6 mg), serine (48.2 mg), threonine (42.2 mg), tryptophan (12.1 mg), tyrosine (2.4 mg), and valine (24.1 mg).

Trace element solution (μg/L): the trace element solution comprised manganese chloride tetrahydrate (MnCl2⋅4H2O, 200 μg), zinc chloride (ZnCl2, 135 μg), iron chloride tetrahydrate (FeCl2⋅4H2O, 30 μg), copper chloride dihydrate (CuCl2⋅2H2O, 15 μg), boric acid (H3BO3, 5 μg), cobalt nitrate hexahydrate [Co(NO3)2⋅6H2O, 30 μg], sodium molybdate dihydrate (Na2MoO4⋅2H2O, 25 μg), and potassium iodate (KIO3, 10 μg).

The medium was prepared by adding 1,000 mL of distilled water, thoroughly mixing all components, and sterilizing at 121°C for 30 min (Camesasca et al., 2018).

Synthetic flour juice (SF) composition:

The SF medium was composed of the following components (per liter): wheat oligopeptides (12 g), magnesium sulfate heptahydrate (MgSO_4_⋅7H_2_O, 0.2 g), manganese sulfate monohydrate (MnSO_4_⋅H_2_O, 0.05 g), potassium dihydrogen phosphate (KH_2_PO_4_, 4 g), dipotassium hydrogen phosphate (K_2_HPO_4_, 4 g), Tween 80 (1 mL), and a vitamin solution (1 mL).

Vitamin solution (mg/L): the vitamin solution contained cobalamin (200 mg), folic acid (200 mg), niacinamide (200 mg), pantothenic acid (200 mg), pyridoxal phosphate (200 mg), and thiamine (200 mg).

Carbohydrate solution (g/L): the carbohydrate solution included glucose (0.5 g), maltose (10 g), fructose (0.5 g), and sucrose (2 g).

The final medium was prepared by adding distilled water to reach a total volume of 1,000 mL, thoroughly mixing all components, and sterilizing at 121°C for 30 min (Vrancken et al., 2008).

### 2.3 Experimental methods

Fresh YPD cultures (1%) were inoculated into 10 mL of SG and SF media. The cultures were incubated at 28°C for 7 days. On days 0 and 7, 200 μL of the cultures were transferred to a 96-well microplate, and absorbance at 600 nm was measured using a Synergy H1 multifunctional microplate reader (BioTek). The initial absorbance (OD_1_) and post-incubation absorbance (OD_2_) were recorded. Calculate fungal biomass ΔOD = (OD_2_–OD_1_)/3 (Zhu et al., 2017).

Tolerance behavior under stressful conditions:

Fresh YPD cultures were inoculated (1% v/v) into YPD media under various stress conditions, including sodium chloride (NaCl), tannin (TA), alcohol (AL), pH, temperature, and sulfur dioxide (SO_2_). The specific stress conditions were as follows:

Sodium chloride (NaCl): 6, 8, and 10% in YPD.

Tannin (TA): 1 g/L, 1.5 g/L, and 2 g/L in YPD.

Alcohol (AL): 12, 14, and 16% in YPD.

pH levels: 3.0, 3.5, 4.0, 5.0, and 7.0 in YPD.

Sodium thiosulfate (S): 0.632 g/L (S-4), 0.1264 g/L (S-8), and 0.1896 g/L (S-12) in YPD.

Cultures were incubated at 28°C for 48 h after inoculation into YPD liquid medium. At both 0 h and 48 h, 200 μL of the culture was transferred into a 96-well plate. Absorbance was measured at 600 nm using a multifunctional plate reader, recording the initial optical density (OD_1_) and the optical density after 48 h (OD_2_). The change in microbial density (ΔOD) was calculated using the formula: (OD_2_–OD_1_)/3.

All experiments were performed in triplicate, and data analysis was conducted using SPSS 26.0 software. One-way ANOVA was employed to assess differences across various sources of isolation, regions, lineages, and substrates, with statistical significance defined as *P* < 0.05. Graphs were created using Origin 2021, while data processing and heatmap generation were performed with MetaboAnalyst.^1^

## 3 Results

### 3.1 Adaptability of diploid *S. cerevisiae* populations

#### 3.1.1 CO_2_ weight loss in SCP and SCJ populations in grape juice

**FIGURE 1:**
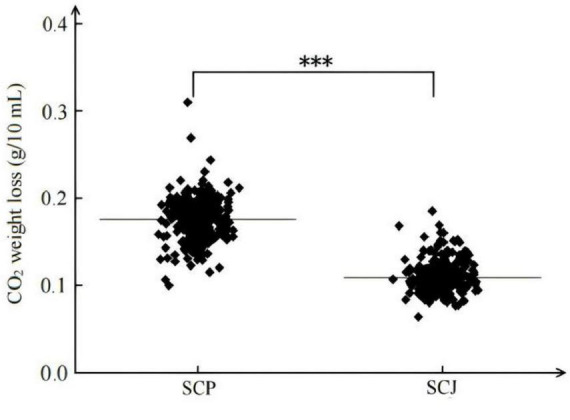
CO_2_ weight loss of SCP and SCJ population in grape juice. ***p < 0.001.

As illustrated in Figure 1, In grape juice, *S. cerevisiae* populations exhibited relatively similar CO_2_ release patterns for SCP or SCJ subpopulation. However, a significant difference was observed between the two subpopulations. The CO_2_ release from the 169-strain SCP population was significantly higher than that from the 138-strain SCJ population (*P* < 0.001).

#### 3.1.2 Adaptation behaviors of SCP and SCJ populations in SG and SF medium

**FIGURE 2:**
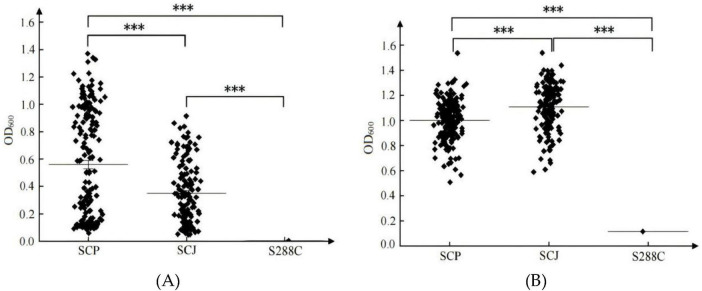
Biomass (OD_600_) of SCP and SCJ population in SG **(A)** and SF **(B)** medium. ***p < 0.001.

The adaptation behaviors of SCP and SCJ *S. cerevisiae* populations differed significantly between the SG and SF media (Figure 2). In SG medium, both populations exhibited highly dispersed biomass distributions with lower mean values (mean < 0.6), whereas in SF medium, the biomass was more concentrated and displayed higher mean values (> 1.0). Specifically, in SG medium, the SCP population demonstrated significantly better growth compared to the SCJ population, while in SF medium, the SCJ population outperformed the SCP population (*P* < 0.001). The S288C model strain, isolated from a spontaneous fermentation environment (Liu and Huang, 2022), exhibited superior growth in SF medium compared to SG medium. These findings indicate that SF medium provides a more favorable environment for the growth of *S. cerevisiae*, whereas the simulated grape juice (SG) medium does not adequately support its growth. Under native or near-native conditions, the SCJ and SCP populations display distinct growth advantages within their respective ecological niches.

#### 3.1.3 Adaptive analysis of XJ and NXJ populations in SG and SF medium

**FIGURE 3:**
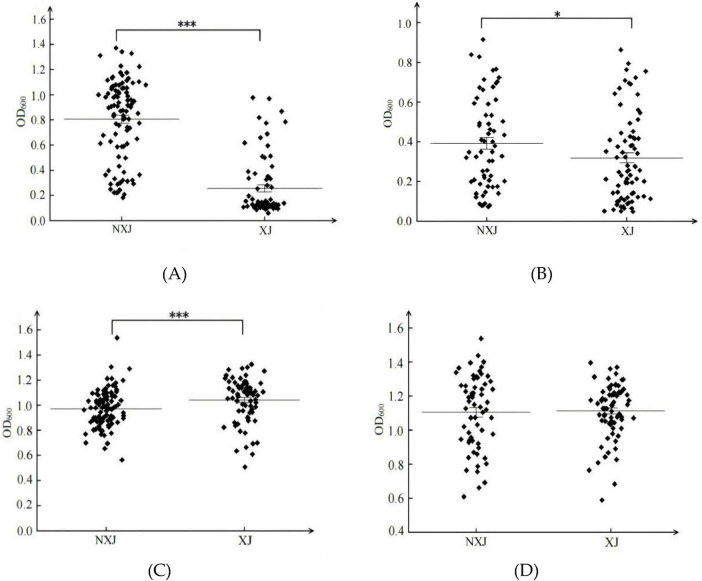
Biomass (OD_600_) of XJ (Xinjiang) and NXJ (no Xinjiang) ***S. cerevisiae*** populations. SCP populations from XJ and NXJ in SG medium **(A)**; SCJ populations from XJ and NXJ in SG medium **(B)**; SCP populations from XJ and NXJ in SF medium **(C)**; SCJ populations from XJ and NXJ in SF medium **(D)**. *p < 0.05, ***p < 0.001.

A total of 149 *S. cerevisiae* strains from the Xinjiang region (XJ) and 158 strains from outside the Xinjiang region (NXJ) were cultured in SG medium (Figures 3A, B). The SCP population demonstrated significantly better growth in NXJ strains compared to XJ strains (Figure 3A), with the average OD_600_ of the NXJ SCP group (0.8) being markedly higher than that of the SCJ groups from both regions (0.3–0.4) (Figure 3B). In SF medium, the SCP population within Xinjiang exhibited significantly higher growth compared to the SCP population outside Xinjiang (Figure 3C). However, no significant difference was observed in the growth characteristics of the SCJ population between the two regions (Figure 3D). The average OD_600_ value of the SCP population within Xinjiang was slightly higher than that of the SCP population outside Xinjiang (Figure 3C).

#### 3.1.4 Adaptation of different lineages of *S. cerevisiae* in SG and SF media

**FIGURE 4:**
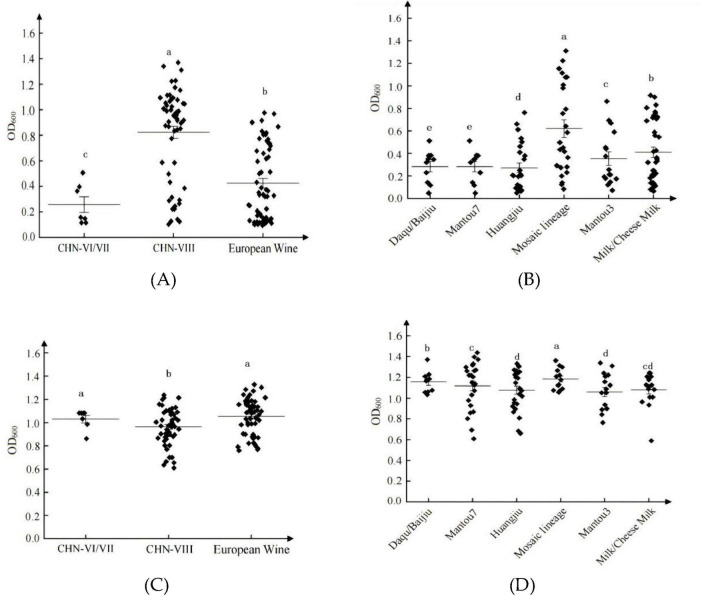
Growth (OD_600_) of different lineages of *S. cerevisiae*. SCP population in SG medium **(A)**; SCJ population in SG medium **(B)**; SCP population in SF medium **(C)**; SCJ population in SF medium **(D)**. a, b, c, d, and e indicate significant differences between groups, the same letters indicate insignificant differences, and different letters indicate significant differences.

In SG medium, significant differences in growth abilities were observed among different lineages within the SCP population (Figure 4A) and the SCJ population (Figure 4B) (*P* < 0.05). The CHN-VIII lineage exhibited the highest growth ability, whereas the CHN-VI/VII lineage showed the weakest adaptation. Within the SCJ population, strains from the Mosaic lineage demonstrated the strongest adaptation capacity.

In SF medium, notable differences in biomass (*P* < 0.05) were also detected among lineages within the SCP population (Figure 4C) and the SCJ population (Figure 4D). Overall, lineages within the SCJ population showed stronger adaptation abilities, with the Mosaic lineage achieving the highest performance.

#### 3.1.5 Tolerance of SCP and SCJ populations under various stresses

**FIGURE 5:**
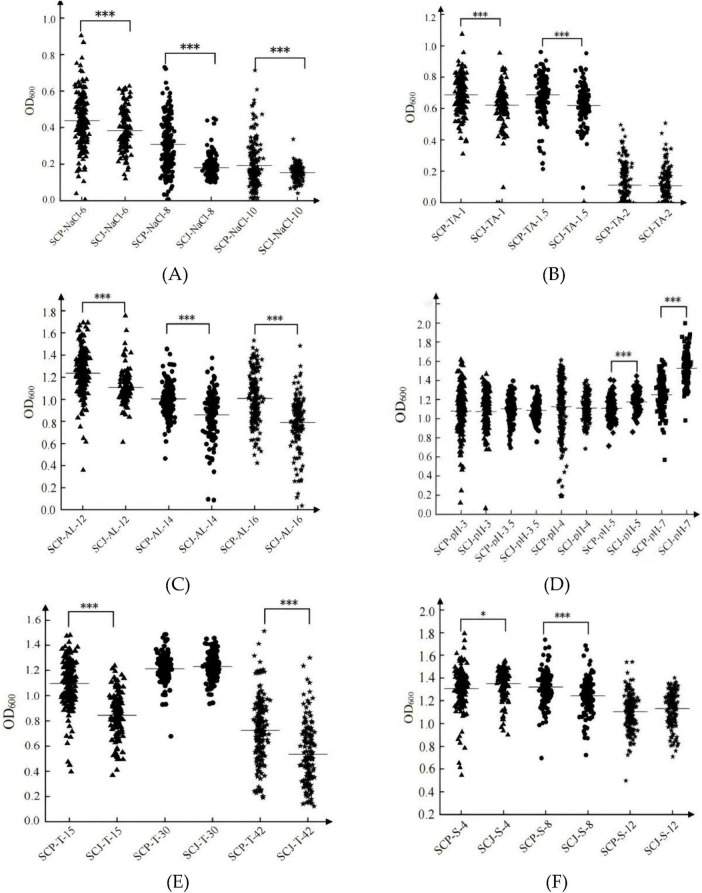
Biomass (OD_600_) of the SCP and SCJ populations under NaCl stress. Biomass of the two groups under NaCl stress **(A)**; Biomass of the two groups under tannin stress **(B)**; Biomass of the two groups under ethanol stress **(C)**; Biomass of the two groups under pH stress **(D)**; Biomass of the two groups under temperature stress **(E)**; Biomass of the two groups under SO_2_ stress **(F)**. *p < 0.05, ***p < 0.001.

Under stress conditions such as 6%–10% NaCl (Figure 5A), 1–1.5 g/L tannin (Figure 5B), 14%–16% ethanol (Figure 5C), extreme temperatures (15°C and 42°C, Figure 5E), and 40–80 mg/L SO_2_ (Figure 5F), the SCP population demonstrated significantly higher adaptability compared to the SCJ population (*P* < 0.05). In contrast, under high pH conditions (pH 5–7), the biomass of the SCJ population was significantly higher than that of the SCP population. However, under low pH conditions (pH 3–4, Figure 5D) and high SO_2_ stress (120 mg/L, Figure 5F), the SCJ population exhibited stronger inhibition. No significant differences in adaptability were observed between the SCP and SCJ populations under these conditions, nor under optimal growth conditions at 30°C (Figure 5E).

### 3.2 Adaptability of representative haploid strains

#### 3.2.1 Adaptability of haploid SCP and SCJ populations

**FIGURE 6:**
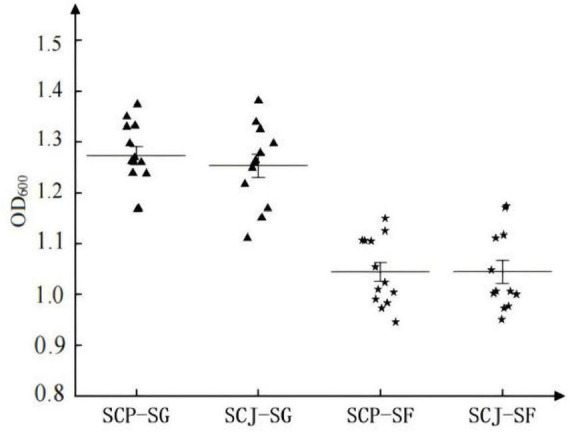
Biomass (OD_600_) of haploid SCP and SCJ population in SG and SF medium.

In a comparison between SG and SF media, the biomass accumulation of haploid SCP and SCJ populations was significantly higher in SG medium than in SF medium (Figure 6). However, within the same medium, no significant difference in biomass accumulation was observed between the haploid SCP and SCJ populations (Figure 6).

#### 3.2.2 Comparison of the tolerance characteristics of haploid and diploid *S. cerevisiae* populations

**FIGURE 7:**
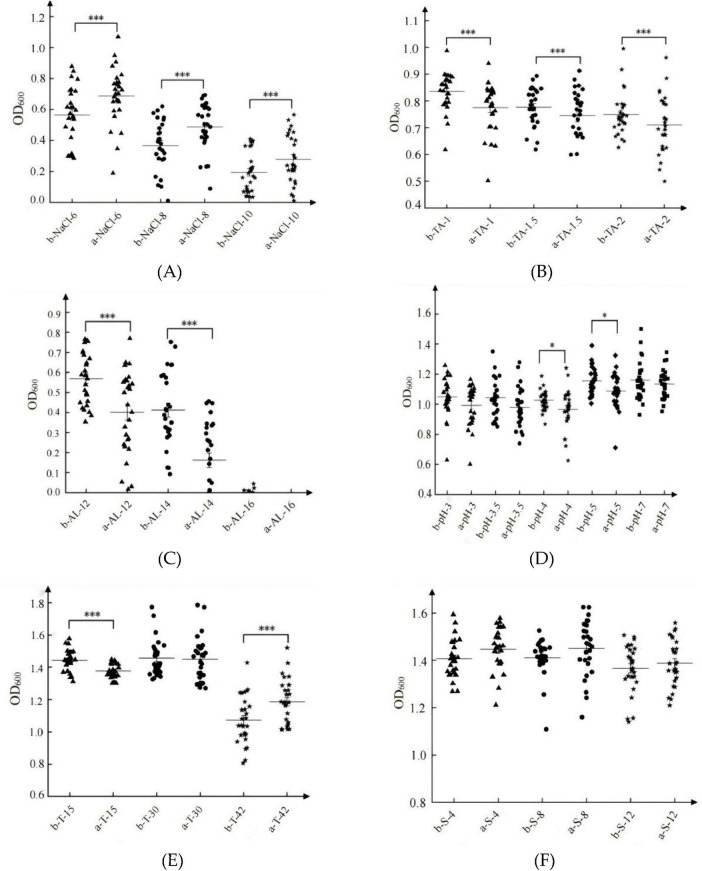
Tolerances of representative haploid and diploid *S. cerevisiae* strains. Biomass of *S. cerevisiae* under NaCl stress **(A)**; Biomass of *S. cerevisiae* under tannin stress **(B)**; Biomass of *S. cerevisiae* under ethanol stress **(C)**; Biomass of *S. cerevisiae* under pH stress **(D)**; Biomass of *S. cerevisiae* under temperature stress **(E)**; Biomass of *S. cerevisiae* under SO_2_ stress **(F)**. In each graph, “a” represents haploid *S. cerevisiae*, and “b” represents diploid *S. cerevisiae*. *p < 0.05, ***p < 0.001.

Under stress conditions, including 6–10 g/L NaCl (Figure 7A), 1–2 g/L tannins (Figure 7B), 12%–14% ethanol (Figure 7C), pH 4–5 (Figure 7D), and low temperatures at 15°C (Figure 7E), diploid *S. cerevisiae* populations demonstrated significantly higher tolerance compared to haploid populations (*P* < 0.05). In contrast, under high-temperature stress at 42°C, haploid populations exhibited significantly greater tolerance than diploid populations (*P* < 0.05) (Figure 7E). For SO_2_ tolerance (40 mg/L–120 mg/L), haploid populations showed slightly higher tolerance than diploid populations (Figure 7F).

The adaptive responses of *S. cerevisiae* populations varied depending on the type of stress, irrespective of ploidy. Among the tested stress factors, ethanol stress exerted the most pronounced impact, with biomass values dropping below 1.0 (OD_600_) and mean values falling below 0.6 (OD_600_) (Figure 7C). Under NaCl stress, while some individual strains achieved biomass values exceeding 1.0, the population mean remained below 0.6 (Figure 7A). Tannin stress resulted in biomass values below 1.0 (OD_600_), with mean values ranging from 0.7 to 0.9 (Figure 7B). Under pH stress, the population biomass mean was approximately 1.0, with relatively low strain variation (Figure 7D). Compared to these stressors, both temperature extremes (Figure 7E) and SO_2_ stress (Figure 7F) had relatively minor effects on the growth of *S. cerevisiae* populations.

#### 3.2.3 Tolerance of haploid and diploid SCP and SCJ *S. cerevisiae* populations

**FIGURE 8:**
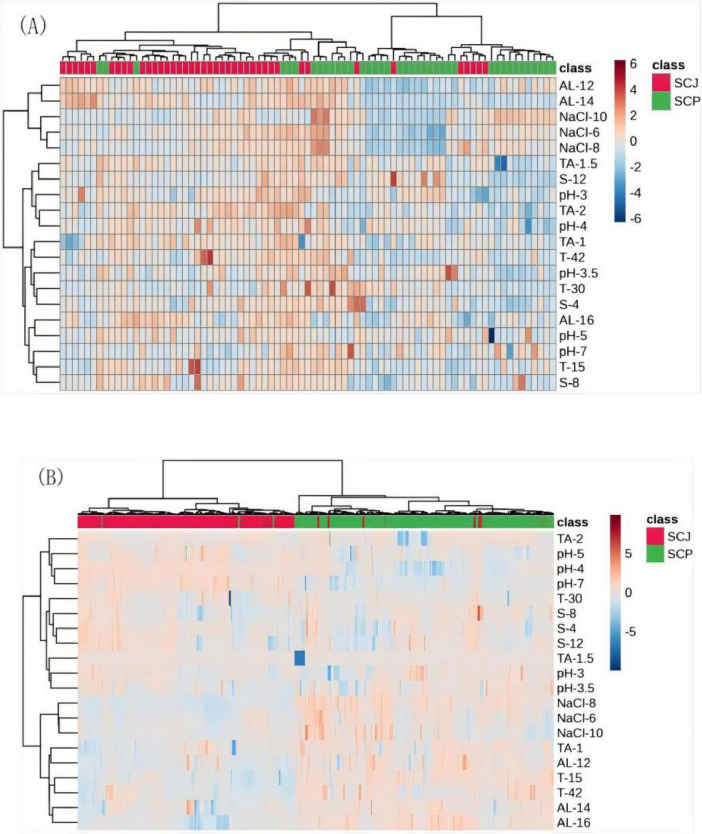
Tolerance heatmap of haploid **(A)** and diploid **(B)** SCP and SCJ *S. cerevisiae* populations.

A comparison of 25 representative haploid strains of *S. cerevisiae* (Figure 8A) and 307 diploid strains (Figure 8B) revealed significant differences in stress tolerance between the SCP and SCJ populations under various stress conditions. Most haploid SCP strains exhibited lower tolerance compared to haploid SCJ strains (Figure 8A). In contrast, the diploid SCP population demonstrated superior tolerance relative to the diploid SCJ population under conditions of high ethanol, low tannins, extreme temperatures (both high and low), high osmotic pressure, and low pH (< 4) (Figure 8B). These results suggest that the diploid SCP population exhibits significantly enhanced tolerance characteristics compared to its haploid counterpart.

## 4 Discussion and conclusion

A comparison of biomass production between the two populations in SG and SF media revealed significantly lower biomass production in SG compared to SF. This suggests that the simulated grape juice medium inhibits *S. cerevisiae* growth and fails to provide an optimal environment for its development (Bagheri et al., 2017; Raas and Dutheil, 2024). In both grape juice and SG media, the SCP population exhibited significantly better growth than the SCJ population. In contrast, the SCJ population demonstrated significantly better adaptability in SF medium than the SCP population. The model strain S288C displayed markedly better growth characteristics in SF medium compared to SG medium, suggesting that *S. cerevisiae* strains from the Wine and Mantou lineages possess distinct growth advantages in their respective native environments, reflecting clear ecological adaptation (Bai et al., 2022). The differences in adaptability between the two populations are associated with specific functional genes and metabolic pathways (Chen and Zhang, 2024). In SG medium, the carbon sources primarily come from glucose and fructose. *S. cerevisiae* may adapt better to the SG medium by upregulating genes related to sugar metabolism, such as the glucose transporter *HXT* (Karri et al., 2024), the glucose kinase gene *GLK1* (Zhang et al., 2024), and the phosphofructokinase gene *PFK1* (Zheng et al., 2024), thereby enabling the SCP population to better thrive in this medium. The simulated dough medium typically contains rich and complex carbon sources. The SCP population may demonstrate enhanced adaptability in such an environment by upregulating genes associated with starch hydrolysis and sugar transport, such as the *STA* gene, which is responsible for breaking down starch into smaller fermentable sugars (Krogerus et al., 2019). genes such as *GPA1*, *SAG1*, and *MAL32*, which are involved in starch degradation and conversion, contribute to enhanced adaptability in starch-rich environments (Dietvorst et al., 2007; Schlarmann et al., 2024; Tamaki, 2007). *S. cerevisiae* adapts to varying environmental pH levels by regulating genes such as the phosphate transporter gene *PHO84* and the proton pump gene *PMA1* (Antunes and Sá-Correia, 2024; Eskes et al., 2018). Grape juice is typically weakly acidic, whereas the pH of dough is closer to neutral or slightly acidic (Bovo et al., 2018; Jayaram et al., 2013). The SCP population has already adapted to this low-pH environment.

In diploid populations, variations in adaptive behavior are observed between different regions and lineages, suggesting that both genetic and environmental factors play a significant role in shaping the adaptive behaviors of *S. cerevisiae* populations (Bai et al., 2022; Legras et al., 2018). Brewing yeasts are classified into distinct phylogenetic lineages based on their geographical origins, including Malaysian, North American, West African, and Wine/European lineages. This classification highlights the combined effects of genetic and geographical factors on the adaptive behavior of brewing yeast populations (Bai et al., 2023). *S. cerevisiae* strains with higher ploidy exhibit improved adaptability (Lahue et al., 2020). Diploid *S. cerevisiae* exhibited greater tolerance and stability compared to haploid populations, consistent with findings from numerous related studies (Harari et al., 2018; Liang and Wang, 2022; Yona et al., 2012). Diploid strains of brewing yeast possess a repertoire of stress tolerance genes, such as *Th2CysPrx*, which significantly enhance their ability to tolerate multiple stressors (Wang et al., 2022), The stress tolerance of diploid *S. cerevisiae* populations under multiple stress factors was found to be stable in the study. Moreover, in the same habitat, diploid yeast populations from SCP habitats demonstrated superior tolerance traits compared to haploid populations. In contrast, haploid populations exhibited instability in their tolerance characteristics, this highlights the key rationale for the widespread distribution of diploid *S. cerevisiae* populations in natural ecosystems.

In stressful environments, the SCP population demonstrates greater tolerance to NaCl, tannin, alcohol, and both low and high temperatures, while the SCJ population exhibits significantly higher biomass in near-neutral environments compared to the SCP population. This is associated with stress acclimatization in their native habitats, driving adaptive evolution in gene expression, metabolic pathways, and stress response mechanisms (Bai et al., 2022). When *S. cerevisiae* is exposed to high osmotic pressure environments (e.g., high salt, high alcohol), the expression of the *GPD1* gene is typically upregulated to help the yeast synthesize more glycerol (Eriksson et al., 2000), maintaining osmotic balance inside and outside the cell. The SCP population enhances its tolerance to low temperatures by upregulating cold-response genes (e.g., *HSP30*) (Sahana et al., 2024), which helps maintain the fluidity and activity of the yeast cell membrane. Additionally, *S. cerevisiae* may upregulate genes such as alcohol dehydrogenase (*ADH1*) and boost lipid metabolism and membrane stability to mitigate alcohol-induced damage to the cell membrane and enzymes (Gutiérrez-Lomelí et al., 2008). In response to tannin stress, the expression of the antioxidant gene *SOD*_1_ may be regulated to mitigate oxidative damage caused by tannin in near-neutral environments (Subramaniyan et al., 2019), the SCJ population outperforms the SCP population, which is related to the slightly acidic nature of the dough, further demonstrating that, under favorable environmental conditions, the best growth environment for *S. cerevisiae* is still its native habitat.

From a laboratory perspective, the phenomena described above clearly indicate that *S. cerevisiae* populations from different habitats exhibit similar population characteristics and demonstrate distinct growth advantages in their native environments. This observation provides theoretical support for the selection of superior strains within populations and offers valuable insights into the development of applications utilizing populations composed of different strains of the same species. Further research is necessary to investigate the relationship between multidimensional stress testing at the strain level and the metabolic activities of these populations, with the goal of optimizing the potential of limited strains in ecological environments.

Current assessments of *S. cerevisiae* adaptability primarily focus on strain selection and practical applications, such as evaluating the fermentation tolerance of *S. cerevisiae* strains isolated from orchards to identify those with superior fermentation performance. Furthermore, individual strains are often evaluated for their adaptability and differentiation potential through laboratory passages. In contrast, this paper adopts a population-level perspective, investigating the ecological adaptive behavior of *S. cerevisiae*. The objective is to provide direct evidence of the species’ remarkable adaptability in ecological environments, while also offering insights from an ecological population perspective to inform the development and application of *S. cerevisiae* strains.

## Data Availability

The raw data supporting the conclusions of this article will be made available by the authors, without undue reservation.

## Footnotes

1
https://www.metaboanalyst.ca
