# Supplementary material for: Comparative adaptability of 307 Saccharomyces cerevisiae strains from winemaking and Mantou fermentation
Source: Front Microbiol. 2025 Apr 11;16:1581370. doi: 10.3389/fmicb.2025.1581370 (PMC12023260; doi:10.3389/fmicb.2025.1581370)
Supplement: Supplementary file 1 [file Table_1.docx]

Table 1: Comparison table of strain, region, isolation source and phylogenetic tree

| number | separately ad | Isolated source | Developmental tree lineage |
| --- | --- | --- | --- |
| GG1-M2 | Gulang County, Gansu | Grape | European Wine |
| GG3-M2 | Gulang County, Gansu | Grape | European Wine |
| GG4-M1 | Gulang County, Gansu | Grape | European Wine |
| GM1-M1 | Minqin County, Gansu | Grape | European Wine |
| GM2-M1 | Minqin County, Gansu | Grape | European Wine |
| GM2-M4 | Minqin County, Gansu | Grape | European Wine |
| GM4-M1 | Minqin County, Gansu | Grape | CHN-VIII |
| GW5-4 | Wuwei City, Gansu | Grape | European Wine |
| GW6-2 | Wuwei City, Gansu | Grape | European Wine |
| GW6-3 | Wuwei City, Gansu | Grape | European Wine |
| HL1-1 | Lankao County, Henan | Grape | CHN-VIII |
| HL1-2 | Lankao County, Henan | Grape | CHN-VIII |
| HL1-3 | Lankao County, Henan | Grape | CHN-VIII |
| HL2-7 | Lankao County, Henan | Grape | CHN-VI/VII |
| HM1-4 | Minquan County, Henan | Grape | CHN-VIII |
| HN1-4 | Ningling County, Henan | Grape | CHN-VIII |
| HN1-5 | Ningling County, Henan | Grape | CHN-VIII |
| HS1-4 | Sui County, Henan | Grape | CHN-VIII |
| HS2-5 | Sui County, Henan | Grape | CHN-VIII |
| HS3-5 | Sui County, Henan | Grape | CHN-VI/VII |
| HS4-1 | Sui County, Henan | Grape | CHN-VI/VII |
| HS5-1 | Sui County, Henan | Grape | CHN-VIII |
| HS6-4 | Sui County, Henan | Grape | CHN-VIII |
| HX1-1 | Xiangcheng County, Henan | Grape | CHN-VI/VII |
| HH1-4 | Zhengzhou, Henan | Grape | CHN-VIII |
| Continued | | | |
| HH4-2 | Zhengzhou, Henan | Grape | CHN-VI/VII |
| HH6-1 | Zhengzhou, Henan | Grape | CHN-VIII |
| HH7-3 | Zhengzhou, Henan | Grape | CHN-VIII |
| MW12-M1 | Wuhai City, Inner Mongolia | Grape | European Wine |
| MW19-M1 | Wuhai City, Inner Mongolia | Grape | European Wine |
| MW22-M1 | Wuhai City, Inner Mongolia | Grape | European Wine |
| MW2-M1 | Wuhai City, Inner Mongolia | Grape | European Wine |
| MW35-M1 | Wuhai City, Inner Mongolia | Grape | European Wine |
| MW36-M1 | Wuhai City, Inner Mongolia | Grape | European Wine |
| MW38-M1 | Wuhai City, Inner Mongolia | Grape | European Wine |
| MW39-M1 | Wuhai City, Inner Mongolia | Grape | European Wine |
| NY1-M2 | Yinchuan City, Ningxia | Grape | CHN-VIII |
| NY1-M4 | Yinchuan City, Ningxia | Grape | CHN-VIII |
| NY4-M1 | Yinchuan City, Ningxia | Grape | CHN-VIII |
| NY4-M2 | Yinchuan City, Ningxia | Grape | CHN-VIII |
| NY4-M5 | Yinchuan City, Ningxia | Grape | CHN-VIII |
| NYN11-M4 | Yongning County, Ningxia | Grape | CHN-VIII |
| NYN12-M1 | Yongning County, Ningxia | Grape | European Wine |
| NYN2-M7 | Yongning County, Ningxia | Grape | CHN-VIII |
| NYN7-M1 | Yongning County, Ningxia | Grape | CHN-VIII |
| NYN8-M4 | Yongning County, Ningxia | Grape | CHN-VIII |
| NYN9-M1 | Yongning County, Ningxia | Grape | CHN-VIII |
| NZ2-M1 | Zhongwei City, Ningxia | Grape | European Wine |
| NZ2-M2 | Zhongwei City, Ningxia | Grape | European Wine |
| NZ3-M1 | Zhongwei City, Ningxia | Grape | European Wine |
| NZ3-M2 | Zhongwei City, Ningxia | Grape | European Wine |
| Continued | | | |
| JQ10-2 | Qingxu County, Shanxi | Grape | CHN-VIII |
| JQ11-1 | Qingxu County, Shanxi | Grape | CHN-VIII |
| JQ6-2 | Qingxu County, Shanxi | Grape | CHN-VIII |
| JQ8-1 | Qingxu County, Shanxi | Grape | CHN-VIII |
| JQ9-1 | Qingxu County, Shanxi | Grape | CHN-VIII |
| JT11-1 | Taigu County, Shanxi | Grape | CHN-VIII |
| JT14-1 | Taigu County, Shanxi | Grape | European Wine |
| JT4-1 | Taigu County, Shanxi | Grape | CHN-VIII |
| JT5-1 | Taigu County, Shanxi | Grape | CHN-VIII |
| JT6-1 | Taigu County, Shanxi | Grape | CHN-VIII |
| JT9-2 | Taigu County, Shanxi | Grape | CHN-VIII |
| JX1-2 | Xia county, Shanxi | Grape | CHN-VIII |
| JX3-1 | Xia county, Shanxi | Grape | CHN-VIII |
| JX3-2 | Xia county, Shanxi | Grape | CHN-VIII |
| JX4-2 | Xia county, Shanxi | Grape | CHN-VIII |
| JX9-1 | Xia county, Shanxi | Grape | CHN-VIII |
| JXN10-1 | Xiangning County, Shanxi | Grape | CHN-VIII |
| JXN13-4 | Xiangning County, Shanxi | Grape | CHN-VIII |
| JXN2 | Xiangning County, Shanxi | Grape | CHN-VIII |
| JXN3-1 | Xiangning County , Shanxi | Grape | CHN-VIII |
| JF1-M1 | Xiangfen County, Shanxi | Grape | CHN-VIII |
| JF3-1 | Xiangfen County, Shanxi | Grape | CHN-VIII |
| JF5-2 | Xiangfen County, Shanxi | Grape | CHN-VIII |
| JF8-4 | Xiangfen County, Shanxi | Grape | CHN-VIII |
| SH13-1 | Huxian county, Shaanxi | Grape | West African cocoa |
| SH5-1 | Huxian county, Shaanxi | Grape | West African cocoa |
| Continued | | | |
| SH6-1 | Huxian county, Shaanxi | Grape | West African cocoa |
| SW1-2 | Pucheng city , Shaanxi | Grape | CHN-VIII |
| SW2-M1 | Pucheng city , Shaanxi | Grape | CHN-VIII |
| SW4-2 | Pucheng city , Shaanxi | Grape | CHN-VIII |
| SW4-3 | Pucheng city , Shaanxi | Grape | CHN-VIII |
| SW6-2 | Pucheng city , Shaanxi | Grape | CHN-VIII |
| SS10-1 | Sanyuan County, Shaanxi | Grape | CHN-VIII |
| SS11-2 | Sanyuan County, Shaanxi | Grape | CHN-VIII |
| SS1-M1 | Sanyuan County, Shaanxi | Grape | CHN-VIII |
| SS3-2 | Sanyuan County, Shaanxi | Grape | CHN-VIII |
| SS6-M4 | Sanyuan County, Shaanxi | Grape | CHN-VIII |
| SAW1-6 | Weiyang District, Xi 'an, Shaanxi | Grape | CHN-VIII |
| SXQ1-3 | Qindu District, Xianyang, Shaanxi | Grape | CHN-VIII |
| SXQ1-5 | Qindu District, Xianyang, Shaanxi | Grape | CHN-VIII |
| SXQ1-8 | Qindu District, Xianyang, Shaanxi | Grape | CHN-VIII |
| SXW3-11 | Weicheng District, Xianyang, Shaanxi | Grape | CHN-VIII |
| SXW4-3 | Weicheng District, Xianyang, Shaanxi | Grape | CHN-VIII |
| SXW5-2 | Weicheng District, Xianyang, Shaanxi | Grape | CHN-VIII |
| SXW7-1 | Weicheng District, Xianyang, Shaanxi | Grape | CHN-VIII |
| 10N1-4 | Aksu Prefecture, Xinjiang | Fermented grape juice | European Wine |
| 37N5-1 | Aksu Prefecture, Xinjiang | Fermented grape juice | European Wine |
| E0-LY4-007 | Aksu Prefecture, Xinjiang | Musalais | European Wine |
| Continued | | | |
| F2-S10-1 | Aksu Prefecture, Xinjiang | Fermented grape juice | CHN-VI/VII |
| G1-3d1-002 | Aksu Prefecture, Xinjiang | Musalais | European Wine |
| F0-K183-2 | Kuqa County, Aksu Prefecture, Xinjiang | Fermented grape juice | Daqu/Baijiu |
| F1-K183-4 | Kuqa County, Aksu Prefecture, Xinjiang | Fermented grape juice | Daqu/Baijiu |
| F1-S209-1 | Shaya, Aksu Prefecture, Xinjiang | Fermented grape juice | Daqu/Baijiu |
| F1-X208-1 | Xinhe,Aksu Prefecture, Xinjiang | Fermented grape juice | Daqu/Baijiu |
| F1-B177-1 | Korla City, Xinjiang | Fermented grape juice | European Wine |
| F1-L185-2 | Luntai, Xinjiang | Fermented grape juice | European Wine |
| F1-Q143-7 | Qiemao County, Bazhou, Xinjiang | Fermented grape juice | Milk/Cheese Milk |
| F2-Q131-7 | Qiemao County, Bazhou, Xinjiang | Fermented grape juice | Milk/Cheese Milk |
| F2-Q142-13 | Qiemao County, Bazhou, Xinjiang | Fermented grape juice | Mantou3 |
| F2-Q142-9 | Qiemao County, Bazhou, Xinjiang | Fermented grape juice | Mantou3 |
| F1-R148-4 | Ruoqiang County, Xinjiang | Fermented grape juice | Milk/Cheese Milk |
| F2-R148-2 | Ruoqiang County, Xinjiang | Fermented grape juice | Milk/Cheese Milk |
| F0-B460-3B | Beitun City, Xinjiang | Fermented grape juice | Mantou7 |
| F2-B465-14 | Beitun City, Xinjiang | Fermented grape juice | European Wine |
| F2-B262-3 | Bo Le, Bozhou, Xinjiang | Fermented grape juice | European Wine |
| F2-B262-5 | Bo Le, Bozhou, Xinjiang | Fermented grape juice | European Wine |
| F2-C320-10 | Changji City, Changji Prefecture,Xinjiang | Fermented grape juice | European Wine |
| F2-C320-13 | Changji City, Changji Prefecture,Xinjiang | Fermented grape juice | European Wine |
| F2-C320-4 | Changji City, Changji Prefecture,Xinjiang | Fermented grape juice | European Wine |
| Continued | | | |
| F2-C320-9 | Changji City, Changji Prefecture,Xinjiang | Fermented grape juice | European Wine |
| F2-C516-13 | Changji City, Changji Prefecture,Xinjiang | Fermented grape juice | European Wine |
| F2-T492-2 | Fukang, Changji Prefecture, Xinjiang | Fermented grape juice | European Wine |
| F2-H324-6 | Hutubi, Changji Prefecture, Xinjiang | Fermented grape juice | European Wine |
| F2-P476-6 | Hutubi, Changji Prefecture, Xinjiang | Fermented grape juice | European Wine |
| F1-M404-3 | Manas, Changji Prefecture, Xinjiang | Fermented grape juice | European Wine |
| F2-X381-11 | Manas, Changji Prefecture, Xinjiang | Fermented grape juice | European Wine |
| F2-M442-2 | Wooden bastions,Changji region, Xinjiang | Fermented grape juice | European Wine |
| F2-M442-3 | Wooden bastions,Changji region, Xinjiang | Fermented grape juice | European Wine |
| F2-Q240-12 | Qitai,Changji region, Xinjiang | Fermented grape juice | European Wine |
| F2-H468-1 | Hami City, Hami Prefecture, Xinjiang | Fermented grape juice | European Wine |
| F2-H469-14 | Hami City, Hami Prefecture, Xinjiang | Fermented grape juice | European Wine |
| F2-H470-11 | Hami City, Hami Prefecture, Xinjiang | Fermented grape juice | European Wine |
| F2-H470-8 | Hami City, Hami Prefecture, Xinjiang | Fermented grape juice | European Wine |
| F1-Y124-3A | Hotan City, Xinjiang | Fermented grape juice | Milk/Cheese Milk |
| F0-C111-62 | Cele County, Hotan City, Xinjiang | Fermented grape juice | European Wine |
| F2-C111-4A | Cele County, Hotan City, Xinjiang | Fermented grape juice | European Wine |
| Continued | | | |
| F0-L104-32 | Luopu County, Hotan City, Xinjiang | Fermented grape juice | CHN-VIII |
| F0-m93-1 | Moyu County, Hotan Prefecture, Xinjiang | Fermented grape juice | CHN-VIII |
| F0-M93-4 | Moyu County, Hotan Prefecture, Xinjiang | Fermented grape juice | CHN-VIII |
| F2-P83-21 | Pishan county, Hotan, Xinjiang | Fermented grape juice | CHN-VIII |
| F2-P85-2 | Pishan county, Hotan, Xinjiang | Fermented grape juice | CHN-VI/VII |
| F2-Y115-X-5 | Yutian County, Hotan, Xinjiang | Fermented grape juice | #N/A |
| F2-A61-4 | Aktao, Kashgar, Xinjiang | Fermented grape juice | CHN-VIII |
| F1-A41-5 | Atush,Kashgar , Xinjiang | Fermented grape juice | CHN-VI/VII |
| F-B12-3 | Bachu ,Kashgar, Xinjiang | Fermented grape juice | CHN-VI/VII |
| F-T8-6 | Tumushuk, Kashgar, Xinjiang | Fermented grape juice | #N/A |
| F2-Y78-1 | Ye City, Kashgar, Xinjiang | Fermented grape juice | West African cocoa |
| F1-Y63-4 | Yingjisha, Kashgar, Xinjiang | Fermented grape juice | #N/A |
| F1-Z76-11 | Kashzep, Xinjiang | Fermented grape juice | West African cocoa |
| F2-S343-9 | Shanshan, Xinjiang | Fermented grape juice | European Wine |
| F2-T346-3 | Tacheng area,Xinjiang | Fermented grape juice | European Wine |
| F2-T431-10 | Tacheng area,Xinjiang | Fermented grape juice | European Wine |
| F2-T431-7 | Tacheng area,Xinjiang | Fermented grape juice | European Wine |
| F2-T438-3 | Tacheng area,Xinjiang | Fermented grape juice | European Wine |
| F2-T450-5 | Tacheng area,Xinjiang | Fermented grape juice | European Wine |
| F2-R252-20 | Shawan, Tacheng area, Xinjiang | Fermented grape juice | European Wine |
| F2-T360-3 | Turpan Prefecture, Xinjiang | Fermented grape juice | European Wine |
| F2-T362-1 | Turpan Prefecture, Xinjiang | Fermented grape juice | European Wine |
| F2-C472-12 | Wujiaqu City, Xinjiang | Fermented grape juice | European Wine |
| F2-W307-11 | Wujiaqu City, Xinjiang | Fermented grape juice | European Wine |
| Continued | | | |
| F2-W307-2 | Wujiaqu City, Xinjiang | Fermented grape juice | European Wine |
| F0-Y336-1 | Ili, Xinjiang | Fermented grape juice | #N/A |
| F2-Y277-5 | Ili, Xinjiang | Fermented grape juice | European Wine |
| F2-Y331-2 | Ili, Xinjiang | Fermented grape juice | European Wine |
| F2-H527-10 | Huocheng, Yili, Xinjiang | Fermented grape juice | European Wine |
| F2-H535-13 | Huocheng, Yili, Xinjiang | Fermented grape juice | European Wine |
| F2-H535-4 | Huocheng, Yili, Xinjiang | Fermented grape juice | European Wine |
| GBH1-J3 | Huining County, Baiyin City, Gansu | Sourdough | Huangjiu |
| GBH1-J4 | Huining County, Baiyin City, Gansu | Sourdough | Huangjiu |
| GBH1-J5 | Huining County, Baiyin City, Gansu | Sourdough | Huangjiu |
| GDL1-J2 | Dingxi City, Gansu | Sourdough | Huangjiu |
| GDL2-J1 | Dingxi City, Gansu | Sourdough | Huangjiu |
| GG1-J1 | Gulang County, Gansu | Sourdough | Mantou7 |
| GG1-J9 | Gulang County, Gansu | Sourdough | Mantou7 |
| GM1-J2 | Minqin County, Gansu | Sourdough | Daqu/Baijiu |
| GM1-J8 | Minqin County, Gansu | Sourdough | Mantou3 |
| GTG5-J1 | Tianshui City, Gansu | Sourdough | Mantou 5 |
| GTG8-J6 | Tianshui City, Gansu | Sourdough | Huangjiu |
| GTG8-J7 | Tianshui City, Gansu | Sourdough | Huangjiu |
| GTG8-J8 | Tianshui City, Gansu | Sourdough | Huangjiu |
| GTG1-J5 | Tianshui City, Gansu | Sourdough | Mantou3 |
| GTG1-J7 | Tianshui City, Gansu | Sourdough | Mantou3 |
| GTG7-J2 | Tianshui City, Gansu | Sourdough | Mantou 5 |
| GTG7-J7 | Tianshui City, Gansu | Sourdough | Mantou 5 |
| GTG2-J1 | Tianshui City, Gansu | Sourdough | Mantou7 |
| GTG2-J5 | Tianshui City, Gansu | Sourdough | Mantou7 |
| Continued | | | |
| GTG2-J7 | Tianshui City, Gansu | Sourdough | Mantou7 |
| GTG2-J8 | Tianshui City, Gansu | Sourdough | Mantou7 |
| GW1-J1 | Wuwei, Gansu | Sourdough | Huangjiu |
| GW1-J3 | Wuwei, Gansu | Sourdough | Huangjiu |
| GZM1-J8 | Minle County, Zhangye City, Gansu | Sourdough | Mantou3 |
| GZM1-J9 | Minle County, Zhangye City, Gansu | Sourdough | Mantou7 |
| HP8-J1 | Pingyu County, Henan | Sourdough | Huangjiu |
| HP8-J2 | Pingyu County, Henan | Sourdough | Huangjiu |
| HP9-J1 | Pingyu County, Henan | Sourdough | Huangjiu |
| HP9-J3 | Pingyu County, Henan | Sourdough | Huangjiu |
| HP9-J4 | Pingyu County, Henan | Sourdough | Huangjiu |
| HP2-J1 | Pingyu County, Henan | Sourdough | Huangjiu |
| HP4-J1 | Pingyu County, Henan | Sourdough | Huangjiu |
| HP4-J5 | Pingyu County, Henan | Sourdough | Daqu/Baijiu |
| HP4-J7 | Pingyu County, Henan | Sourdough | Huangjiu |
| HP10-J3 | Pingyu County, Henan | Sourdough | Mantou7 |
| HP3-J12 | Pingyu County, Henan | Sourdough | Daqu/Baijiu |
| HP3-J6 | Pingyu County, Henan | Sourdough | Daqu/Baijiu |
| HP1-J5 | Pingyu County, Henan | Sourdough | Huangjiu |
| HP1-J6 | Pingyu County, Henan | Sourdough | Huangjiu |
| HR1-J3 | Runan County, Henan | Sourdough | Mantou3 |
| HS1-Q2 | Shangcai County, Henan | Sourdough | Daqu/Baijiu |
| HX1-J1 | Xiangcheng County, Henan | Sourdough | Daqu/Baijiu |
| XY1-J2 | Yuncheng, Shanxi | Sourdough | Mantou7 |
| XY1-J3 | Yuncheng, Shanxi | Sourdough | Mantou7 |
| XY2-J3 | Yuncheng, Shanxi | Sourdough | Mantou7 |
| Continued | | | |
| XY2-J4 | Yuncheng, Shanxi | Sourdough | Mantou7 |
| XY3-J1 | Yuncheng, Shanxi | Sourdough | Mantou7 |
| XY3-J7 | Yuncheng, Shanxi | Sourdough | Mantou7 |
| XY3-J8 | Yuncheng, Shanxi | Sourdough | Mantou7 |
| SAY3-J3 | Yanta District, Xi 'an, Shaanxi | Sourdough | Mantou7 |
| SAY3-J7 | Yanta District, Xi 'an, Shaanxi | Sourdough | Mantou7 |
| SAY4-J1 | Yanta District, Xi 'an, Shaanxi | Sourdough | Mantou7 |
| SAY4-J3 | Yanta District, Xi 'an, Shaanxi | Sourdough | Mantou7 |
| SAY4-J8 | Yanta District, Xi 'an, Shaanxi | Sourdough | Mantou1/Mantou 2 |
| SXC1-J2 | Xianyang, Shaanxi | Sourdough | Mantou3 |
| SXC1-J3 | Xianyang, Shaanxi | Sourdough | Mantou3 |
| SXC1-J8 | Xianyang, Shaanxi | Sourdough | Mantou3 |
| SY1-J1 | Yangling in Shaanxi | Sourdough | Mantou7 |
| SY1-J2 | Yangling in Shaanxi | Sourdough | Mantou7 |
| SY1-J3 | Yangling in Shaanxi | Sourdough | Mantou7 |
| SY1-J5 | Yangling in Shaanxi | Sourdough | Mantou7 |
| SY1-J8 | Yangling in Shaanxi | Sourdough | Mantou7 |
| JAK1-J1 | Keping County, Aksu, Xinjiang | Sourdough | Milk/Cheese Milk |
| JAK1-J3 | Keping County, Aksu, Xinjiang | Sourdough | Milk/Cheese Milk |
| JAK3-J1 | Keping County, Aksu, Xinjiang | Sourdough | Mosaic lineage |
| JAK5-J1 | Keping County, Aksu, Xinjiang | Sourdough | Daqu/Baijiu |
| JAK5-J2 | Keping County, Aksu, Xinjiang | Sourdough | Huangjiu |
| JAS5-J1 | Shaya County, Aksu, Xinjiang | Sourdough | Huangjiu |
| JAS5-J3 | Shaya County, Aksu, Xinjiang | Sourdough | Mantou3 |
| JAW1-J4 | Wushi County, Aksu, Xinjiang | Sourdough | Daqu/Baijiu |
| JAX1-J1 | Xinhe County, Aksu, Xinjiang | Sourdough | Daqu/Baijiu |
| Continued | | | |
| JKT2-J1 | Aktao, Xinjiang | Sourdough | Milk/Cheese Milk |
| JKT2-J3 | Aktao, Xinjiang | Sourdough | Mantou7 |
| JKT2-J7 | Aktao, Xinjiang | Sourdough | Daqu/Baijiu |
| JD1-J1 | Aral City, Xinjiang | Sourdough | Huangjiu |
| JT1-J10 | Atushi,Xinjiang | Sourdough | Mantou3 |
| JT1-J3 | Atushi,Xinjiang | Sourdough | Huangjiu |
| JT1-J1 | Atushi,Xinjiang | Sourdough | Huangjiu |
| JH1-J2 | Hami, Xinjiang | Sourdough | Daqu/Baijiu |
| JH2-J1 | Hami, Xinjiang | Sourdough | Mantou7 |
| JH2-J4 | Hami, Xinjiang | Sourdough | Mantou7 |
| JH4-J1 | Hotan,Xinjiang | Sourdough | Mantou3 |
| JH5-J5 | Hotan,Xinjiang | Sourdough | Mantou3 |
| JH6-J3 | Hotan,Xinjiang | Sourdough | Daqu/Baijiu |
| JHP1-J1 | Pishan county, Hotan, Xinjiang | Sourdough | Mosaic lineage |
| JHP1-J4 | Pishan county, Hotan, Xinjiang | Sourdough | Mosaic lineage |
| JK5-J3 | Kashgar, Xinjiang | Sourdough | Daqu/Baijiu |
| JK5-J6 | Kashgar, Xinjiang | Sourdough | Huangjiu |
| JKA5-J2 | Kashgar Atush, Xinjiang | Sourdough | Mosaic lineage |
| JKA6-J1 | Kashgar Atush, Xinjiang | Sourdough | Mosaic lineage |
| JKA6-J11 | Kashgar Atush, Xinjiang | Sourdough | Mosaic lineage |
| JKA6-J6 | Kashgar Atush, Xinjiang | Sourdough | Mosaic lineage |
| JKA6-J7 | Kashgar Atush, Xinjiang | Sourdough | Mosaic lineage |
| JKA6-J8 | Kashgar Atush, Xinjiang | Sourdough | Mosaic lineage |
| JKB1-J4 | Kashbachu County, Xinjiang | Sourdough | Milk/Cheese Milk |
| JKJ1-J5 | Jiashi County, Kashgar, Xinjiang | Sourdough | Mantou3 |
| JKJ5-J7 | Jiashi County, Kashgar, Xinjiang | Sourdough | Huangjiu |
| Continued | | | |
| JKJ5-J8 | Jiashi County, Kashgar, Xinjiang | Sourdough | Huangjiu |
| JKJ8-J1 | Jiashi County, Kashgar, Xinjiang | Sourdough | Mosaic lineage |
| JKJ8-J2 | Jiashi County, Kashgar, Xinjiang | Sourdough | Mosaic lineage |
| JKSF1-J1 | Tiafu County, Kashgar, Xinjiang | Sourdough | Daqu/Baijiu |
| JKSF1-J4 | Tiafu County, Kashgar, Xinjiang | Sourdough | Milk/Cheese Milk |
| JKSF1-J7 | Tiafu County, Kashgar, Xinjiang | Sourdough | Mosaic lineage |
| JKSF1-J8 | Tiafu County, Kashgar, Xinjiang | Sourdough | Milk/Cheese Milk |
| JKM1-J2 | Meghati County, Kashgar, Xinjiang | Sourdough | Milk/Cheese Milk |
| JKM1-J3 | Meghati County, Kashgar, Xinjiang | Sourdough | Milk/Cheese Milk |
| JKM1-J7 | Meghati County, Kashgar, Xinjiang | Sourdough | Huangjiu |
| JSC1-J2 | Shache, Kashgar, Xinjiang | Sourdough | Mantou3 |
| JSC1-J3 | Shache, Kashgar, Xinjiang | Sourdough | Mantou3 |
| JSC1-J5 | Shache, Kashgar, Xinjiang | Sourdough | Mantou3 |
| JSC3-J2 | Shache, Kashgar, Xinjiang | Sourdough | Mantou3 |
| JSC3-J3 | Shache, Kashgar, Xinjiang | Sourdough | Mosaic lineage |
| JHP2-J4 | Keping County, Xinjiang | Sourdough | Mantou3 |
| JKE1-J2 | Korla, Xinjiang | Sourdough | Daqu/Baijiu |
| JKE1-J4 | Korla, Xinjiang | Sourdough | #N/A |
| JKE1-J6 | Korla, Xinjiang | Sourdough | Milk/Cheese Milk |
| JKER1-J1 | Ruoqiang County, Xinjiang | Sourdough | Daqu/Baijiu |
| JKER1-J2 | Ruoqiang County, Xinjiang | Sourdough | Daqu/Baijiu |
| JKER1-J5 | Ruoqiang County, Xinjiang | Sourdough | Milk/Cheese Milk |
| JS10-J6 | Shihezi City, Xinjiang | Sourdough | Daqu/Baijiu |
| JS1-J4 | Shihezi City, Xinjiang | Sourdough | Mantou7 |
| JS2-J1 | Shihezi City, Xinjiang | Sourdough | Mantou7 |
| JS3-J4 | Shihezi City, Xinjiang | Sourdough | Daqu/Baijiu |
| Continued | | | |
| JS4-J2 | Shihezi City, Xinjiang | Sourdough | Daqu/Baijiu |
| JS8-J1 | Shihezi City, Xinjiang | Sourdough | Huangjiu |
| JS8-J4 | Shihezi City, Xinjiang | Sourdough | Daqu/Baijiu |
| JYG2-J3 | Gongliu County, Yili, Xinjiang | Sourdough | Huangjiu |
| JYG2-J8 | Gongliu County, Yili, Xinjiang | Sourdough | Huangjiu |
| JYX1-J10 | Xinyuan Yili, Xinjiang | Sourdough | Milk/Cheese Milk |
| JYX1-J2 | Xinyuan Yili, Xinjiang | Sourdough | Milk/Cheese Milk |
| JYY1-J5 | Yili Yining, Xinjiang | Sourdough | Milk/Cheese Milk |
| JYZ1-J11 | Zhaosu, Yili, Xinjiang | Sourdough | Milk/Cheese Milk |
| JYZ1-J2 | Zhaosu, Yili, Xinjiang | Sourdough | Milk/Cheese Milk |
| JYZ1-J4 | Zhaosu, Yili, Xinjiang | Sourdough | Milk/Cheese Milk |
| JYZ1-J5 | Zhaosu, Yili, Xinjiang | Sourdough | Milk/Cheese Milk |
| JYZ1-J6 | Zhaosu, Yili, Xinjiang | Sourdough | Milk/Cheese Milk |
| JYZ1-J8 | Zhaosu, Yili, Xinjiang | Sourdough | Milk/Cheese Milk |
| JY1-J1 | Yili Yining, Xinjiang | Sourdough | Mantou3 |
